# Murine hematopoietic stem cell reconstitution potential is maintained by osteopontin during aging

**DOI:** 10.1038/s41598-018-21324-x

**Published:** 2018-02-12

**Authors:** Jin Li, Carmen Carrillo García, Tamara Riedt, Maria Brandes, Sabrina Szczepanski, Peter Brossart, Wolfgang Wagner, Viktor Janzen

**Affiliations:** 1 Department of Internal Medicine III, Division of Hematology/Oncology, University of Bonn, Bonn, Germany; 2 Helmholtz-Institute for Biomedical Engineering, RWTH Aachen University Medical School, Aachen, Germany

## Abstract

In adult mammals, hematopoietic stem cells (HSCs) reside in the bone marrow and are in part regulated by the bone marrow microenvironment, called the stem cell niche. We have previously identified the bone marrow morphogen osteopontin (OPN), which is abundantly present in the bone marrow extracellular matrix, as a negative regulator of the size of the HSC pool under physiological conditions. Here, we study the impact of OPN on HSC function during aging using an OPN-knockout mouse model. We show that during aging OPN deficiency is associated with an increase in lymphocytes and a decline in erythrocytes in peripheral blood. In a bone marrow transplantation setting, aged OPN-deficient stem cells show reduced reconstitution ability likely due to insufficient differentiation of HSCs into more mature cells. In serial bone marrow transplantation, aged OPN^−/−^ bone marrow cells fail to adequately reconstitute red blood cells and platelets, resulting in severe anemia and thrombocytopenia as well as premature deaths of recipient mice. Thus, OPN has different effects on HSCs in aged and young animals and is particularly important to maintain stem cell function in aging mice.

## Introduction

In mammalian tissues that undergo high cell turnover, such as hematopoietic system, a small population of stem cells maintains organ regeneration throughout the animal’s life span. However, the functionality of stem cells declines during aging and can contribute to aging-associated impairments in tissue regeneration^1^. In the hematopoietic system, numerous pathophysiological changes become evident with age, including compromised immune competence, anemia, and increased incidence of myeloid cancers^2^. Accumulating evidence indicates that aged hematopoietic stem cells (HSCs) increase in number due to a higher rate of self-renewal cell divisions while displaying reduced intrinsic reconstitution ability^3–7^. DNA damage, epigenetic dysregulation, and clonal selection are related to the altered functional capacity of HSCs during aging^5,8–10^. In addition, extrinsic modulators, such as components of the stem cell niche, affect the aging process of HSCs^11,12^. Changes in levels of cytokines, such as TGF-β^13^, or the cellular composition of the bone marrow^14^ have been associated with HSC aging.

The phosphorylated glycoprotein osteopontin (OPN) is an extracellular matrix component of the bone marrow with important roles in tissue homeostasis, inflammatory responses, and tumor metastasis^15^. The expression of OPN within the bone marrow is highly restricted to the endosteal surface^16,17^, a location where HSCs have been found to reside preferentially^18^. OPN binds to cells through integrins or the CD44 receptor, subsequently activating multiple signaling pathways. When HSCs are transplanted into wild-type (WT) or OPN^−/−^ mice, they exhibit aberrant attachment and engraftment^17^, suggesting the dependence of HSCs on OPN in these processes. Moreover, OPN deficiency within the bone marrow microenvironment results in an increase in primitive HSC numbers^16^. A recent study showed that specimens of OPN-expressing cells displayed more evidence of aplastic anemia than did chronic myeloid leukemia specimens, suggesting that changes in the components of the bone marrow microenvironment contribute to impaired hematopoiesis^19^. Similar changes in OPN expression have been reported in different non-hematopoietic tissues in age-associated diseases, such as vascular calcification^20^ and neurodegeneration^21^. Moreover, an age-dependent shift from osteogenesis to adipogenesis during the differentiation of mesenchymal stromal stem cells has been associated with a decrease in OPN expression in aged rodents^22^. More recently has been reported that osteopontin exposure to aged HSC can attenuate their aging-associated phenotype^23^.

In this study, we analyze the impact of OPN deficiency on HSC function in aged mice using an OPN-knockout mouse model. We show that OPN deficiency was associated with changes in peripheral blood cell counts compared to WT controls, starting at the age of 12 months. We demonstrate that in the absence of OPN, HSCs possess a significantly reduced ability to reconstitute multiple hematopoietic lineages. In serial bone marrow transplants, OPN^−/−^ stem cells are unable to sustain hematopoietic reconstitution beyond the second round of transplantation, resulting in deaths of recipients. In contrast to the known roles of OPN in young mice our data demonstrate different roles of OPN in aged and young animals.

## Results

### OPN deficiency affects lymphopoiesis and erythropoiesis in aged mice

**Figure 1:**
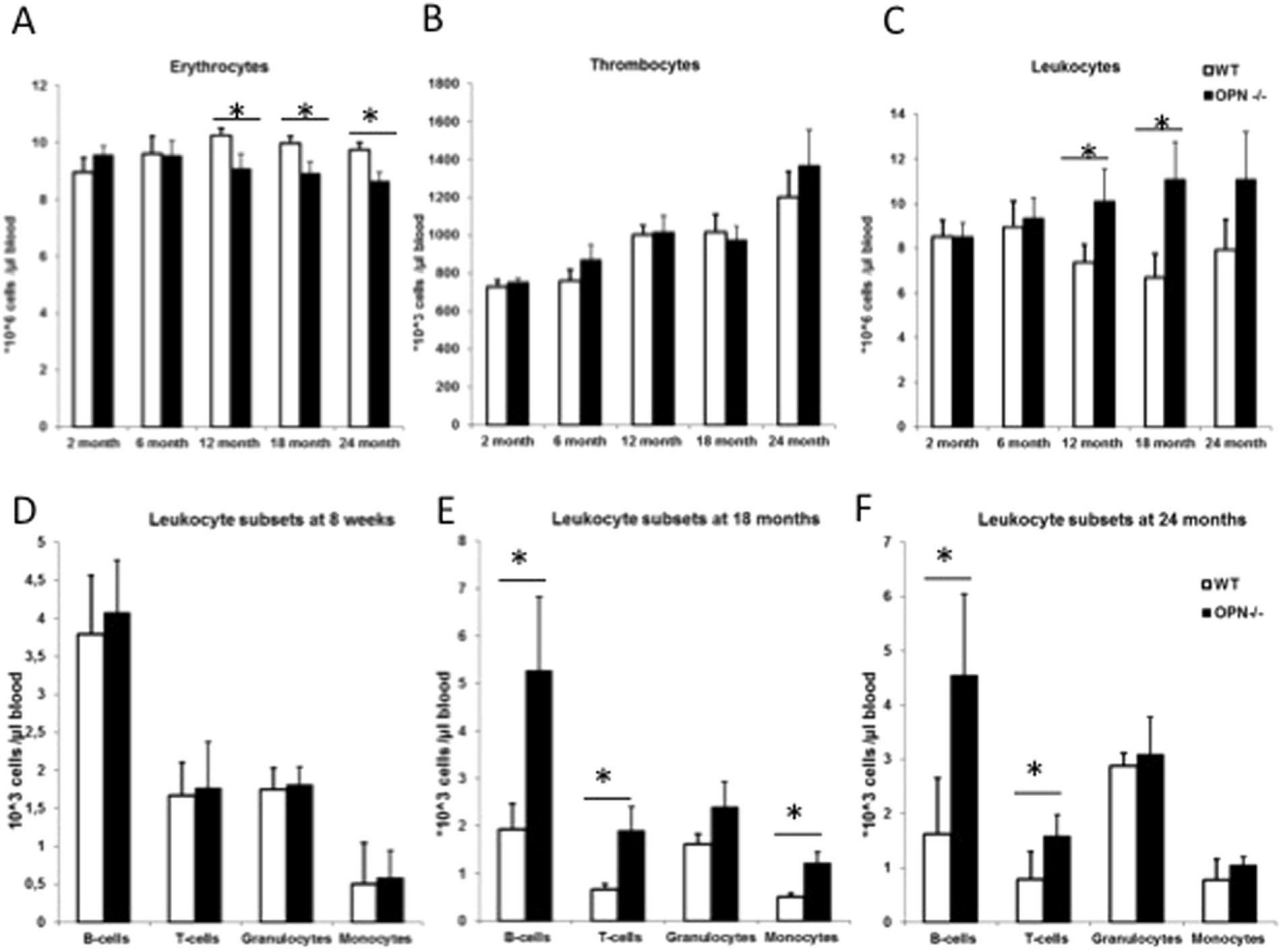
OPN deficiency alters peripheral blood cell counts in aged mice. (**A**–**C**) Cell counts of erythrocytes, thrombocytes, and leukocytes respectively in young and aged mice. (**D**–**F**) Cell counts of leukocyte subtypes defined by surface marker expression using flow cytometry in young (**D**), 18 month (**E**) and 24 month (**F**) old mice. Values are the mean ± SEM; n ≥ 12; *P ≤ 0.05.

To establish the role of osteopontin in HSC aging, we first assayed the changes of OPN mRNA expression in total tibia lysates of young and old wild-type mice. We detected a significant reduction in OPN-mRNA levels in bones of 20-month-old mice compared to 2 month old animals (Supplementary Fig. 1A). This data is consistent with a recent report showing reduced OPN protein concentration in bone marrow fluid in old mice compared to young^23^. Next we analyzed the impact of OPN deficiency on hematopoiesis during the course of animals aging. We did not observe any differences in mature blood cell subtypes in young animals (Fig. 1A–C), in accordance with previous reports^16,17^. Mice from both genotypes (e.g. OPN^−/−^ and WT) showed comparable body sizes at the advanced age of 18 and 24 month (Supplementary Fig. 1B). Analyses of peripheral blood cell counts revealed a significantly reduced number of red blood cells in aged OPN^−/−^ animals compared to age-matched controls (Fig. 1A), with no significant differences in platelet counts between the genotypes (Fig. 1B). In addition, aged OPN-deficient mice had a higher number of leukocytes at 12 and 18 month of age compared to WT controls (Fig. 1C). A differential analysis of white blood cells (WBCs) showed no differences between WBC subtypes in young animals (Fig. 1D) but a significant increase in both B and T lymphocytes in aged OPN^−/−^ mice compared to age-matched controls (Fig. 1E,F), making these cell populations accountable for the increase in total leukocyte counts.

**Figure 2:**
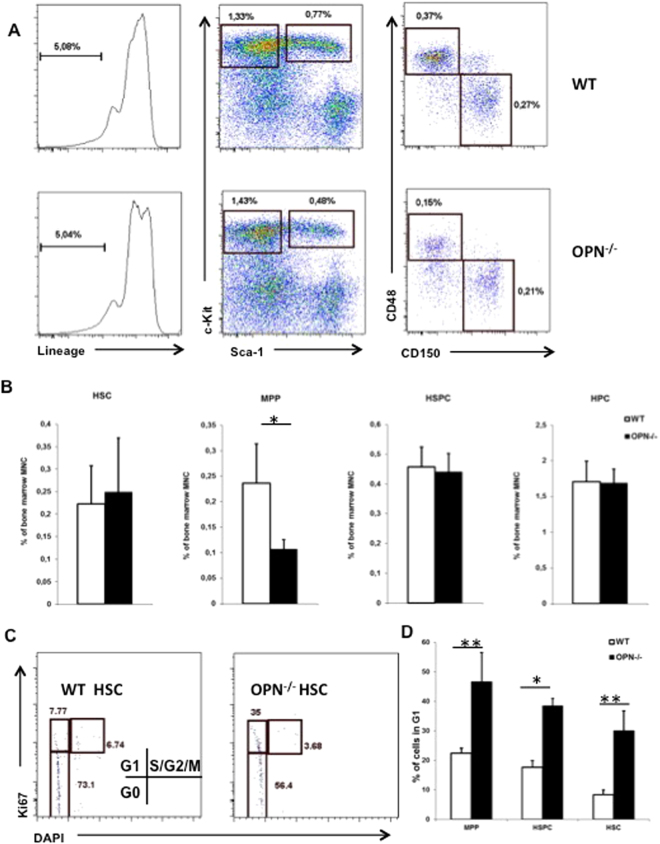
OPN alters the composition of the primitive HSC compartment. (**A**) Representative plots from 22-month-old WT and OPN^−/−^ bone marrow cells. (**B**) Cumulative data of HSPC subpopulations calculated as percentage of total bone marrow mononuclear cells. Values are the mean ± SD; n = 7; *P ≤ 0.05. (**C**) Representative plots of cell cycle analyses by Ki67 and DAPI gated on HSC population. (**D**) Cumulative analyses of the proportion of stem and progenitor cells in G1 phase of the cell cycle in WT and OPN−/− mice. Values are the mean ± SD; n = 3; *P ≤ 0.05, **P ≤ 0.01.

Next, we analyzed the effects of OPN deficiency on the composition of bone marrow cell subtypes. There was no difference in bone marrow cellularity between aged WT and OPN^−/−^ mice (Supplementary Fig. 2A). We observed no significant changes in the numbers of HSPC subpopulation (Lin^−^cKit^+^Sca1^+^), a highly enriched HSC population (Lin^−^cKit^+^Sca1^+^CD48^−^CD150^+^), or myeloid-restricted progenitor cells (Lin^−^cKit^+^Sca1^−^). The only significant difference observed between aged OPN^−/−^ and age-matched controls was the number of multipotent progenitor (MPP) cells (Lin^−^cKit^+^Sca1^+^CD48^+^CD150^−^), which decreased in OPN^−/−^mice (Fig. 2A and B).

Given that OPN influences the rates of apoptosis and proliferation in the primitive HSC compartment of young mice^16,17^, we questioned whether the same effects of OPN deficiency exist in aged animals. First, we analyzed whether OPN deficiency affects the cell division rates of primitive HSCs in both young and aged OPN^−/−^ and WT mice. Using a BrdU incorporation assay, we determined the numbers of HSPCs (Lin^−^cKit^+^Sca1^+^) that initiated at least one cell division within 24 h of BrdU administration. Consistent with previous reports^17^, HSPCs from young OPN^−/−^ mice displayed a significantly higher proliferation rate than their WT counterparts (Supplementary Fig. 2B); no differences in the frequency of cell divisions within the primitive bone marrow subpopulations between WT and OPN^−/−^ mice were observed (Supplementary Fig. 2C). Next we analyzed the cell cycle state of primitive bone marrow subsets, as long term repopulating HSC are mostly persisting in the G0 state of the cell cycle^24^, while activated HSC are found in G1 phase. Using Ki67-DAPI assay we found a shift from G0 toward G1 state in OPN-deficient HSC compared to WT counterpart (Fig. 2C,D). Finally, using the annexin V/DAPI assay, we analyzed freshly isolated bone marrow cells for annexin V expression and exclusion of the nuclear dye DAPI. No significant differences in the numbers of annexin V^+^/DAPI^−^ cells within the HSPC-containing populations were detectable (Supplementary Fig. 2D).

### Osteopontin deficiency affects serial transplantation ability of aged HSCs

**Figure 3:**
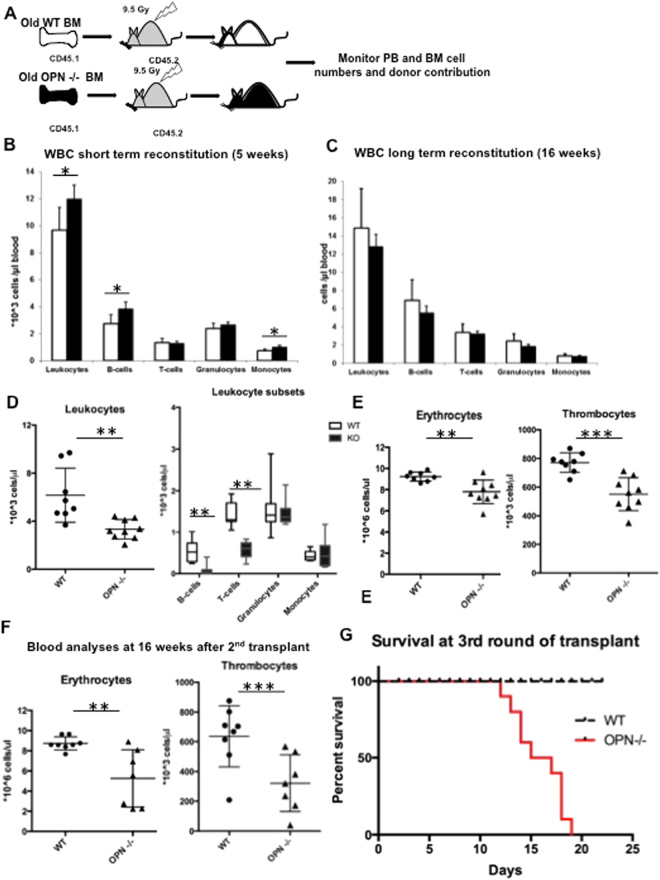
OPN deficiency results in loss of serial reconstitution ability in aged animals. (**A**) Schematic of the transplantation and analysis setting. Bone marrow from CD45.1-bearing WT or OPN-KO mice was transplanted into lethally irradiated CD45.2-bearing recipients and monitored for peripheral blood and bone marrow reconstitution. (**B**) Short- and (**C**) long-term analysis of peripheral blood cells at 5 and 16 weeks after transplantation. (**D**) Total leukocyte count and leukocyte subsets from a secondary bone marrow transplant. (**E**) Red blood cell and platelet counts in the secondary recipients of aged OPN−/− and WT bone marrow 10 weeks after bone marrow transplant. (**F**) Analysis of blood cell counts of the same mice 16 weeks after the second round of bone marrow transplant. (**G**) Kaplan Meier plot indicating survival after third round of bone marrow transplant. Values are the mean ± SD, n ≥ 7, *P < 0.05, **P < 0.01, ***P < 0.001.

To examine whether OPN deficiency in aged mice impacts the reconstitution ability of lethally irradiated mice, we transplanted OPN^−/−^ and WT bone marrow cells from 22-month-old mice into lethally irradiated WT recipients and monitored their peripheral blood and bone marrow reconstitution ability. A congenic mouse system was utilized: donor cells expressed the pan-hematopoietic surface marker CD45.1 and were transplanted into CD45.2-bearing hosts (Fig. 3A). Although we noticed significantly faster recovery of B cell and monocyte lineages at early time points (5 weeks after transplantation) (Fig. 3B and Supplementary Fig. 3A), no changes in peripheral blood cell subtypes were observed at later time points (16 weeks after transplantation) (Fig. 3C and Supplementary Fig. 3B). Analyses of bone marrow lineages, however, revealed a significant reduction in cells of the erythroid lineage, whereas no differences in granulocytic or lymphoid lineages were detectable (Supplementary Fig. 3C).

Several proteins involved in cell cycle control have been shown to be essential for the preservation of the reconstitution ability of HSCs^6,25,26^. To analyze whether OPN deletion in aged mice influences the maintenance of the stem cell pool, we performed serial bone marrow transplantation. Although we did not observe altered long-term engraftment after the primary transplant, we noted a significant delay in WBC reconstitution 10 weeks after the second round of transplantation (Fig. 3D) associated with insufficient lymphocyte recovery (Fig. 3E). Most striking, however, was the inferior reconstitution of red blood cells and thrombocytes in mice receiving bone marrow from aged OPN^−/−^ donors, resulting in severe anemia and thrombocytopenia at 16 weeks post-transplant (Fig. 3E). About 50% of OPN^−/−^ bone marrow recipients died around 4 month after transplant, while all WT bone marrow recipients survived until 7 month after transplant.

Twelve weeks after the second round of transplantation, a third transplant round was performed. While recipients of aged WT bone marrow showed sufficient hematopoietic reconstitution, none of the recipients of aged OPN^−/−^ bone marrow survived beyond 3 weeks after transplant (Fig. 3G); death was therefore presumed to be from hematopoietic failure. When we analyzed recipient bone marrow after the second round of transplantation, we did not see a loss of immunophenotypically defined stem cells (Supplementary Fig. 3D). These data indicate that OPN deficiency not only results in impaired reconstitution of lymphoid lineages in a repetitive transplant but also, to an even greater extent, in impaired erythroid and thrombopoietic reconstitution. These findings are consistent with a loss of reconstitution ability of HSCs in the absence of OPN in aged animals.

### OPN-deficiency perturbs differentiation of aged stem and progenitor cells in a competitive transplant setting

**Figure 4:**
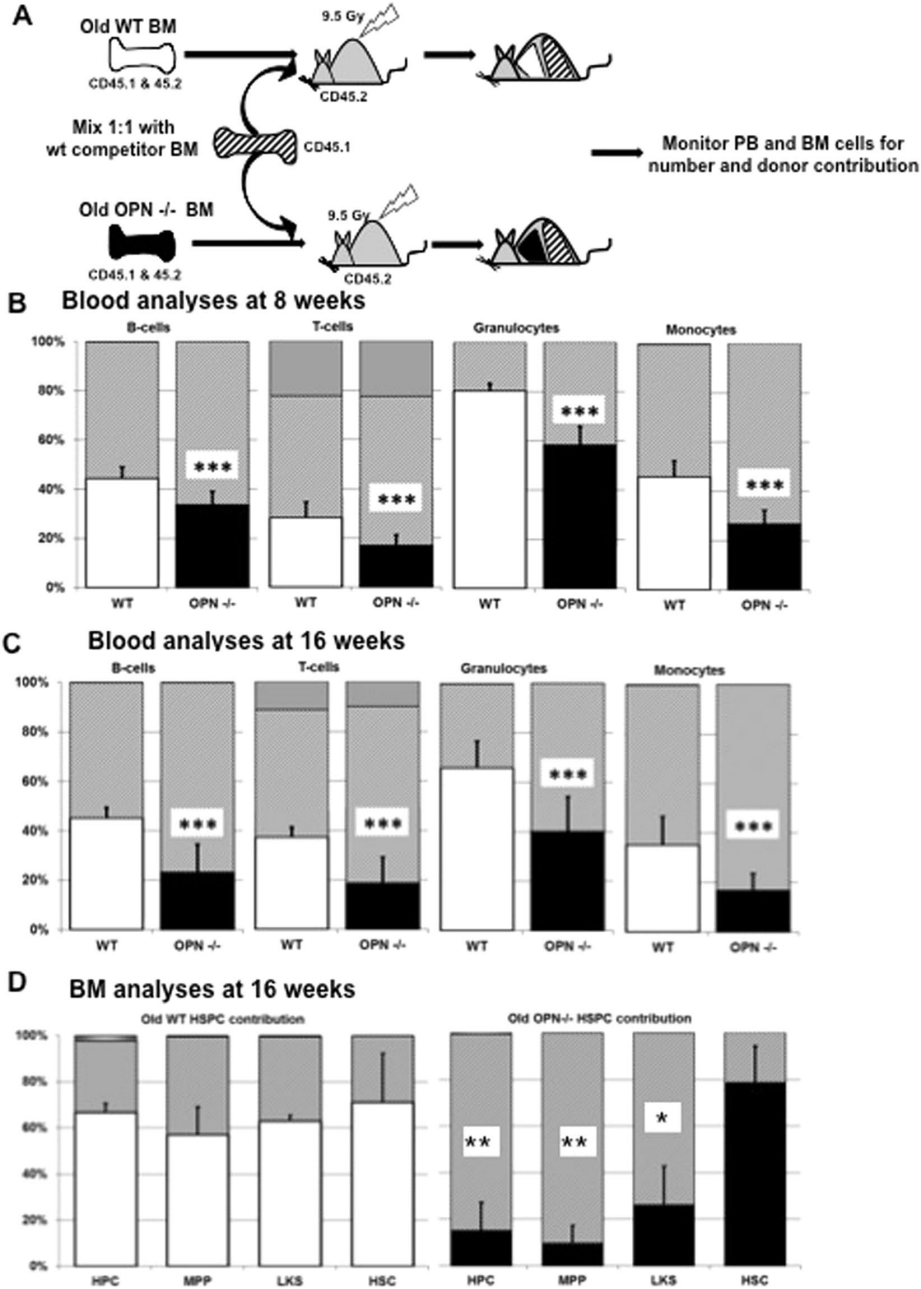
Aged OPN-deficient bone marrow cells display reduced reconstitution ability in a competitive transplantation setting. (**A**) Schematic of the competitive repopulation assay, which was performed to test the ability of aged OPN mutant stem cells to compete against WT HSCs. Analyses of the peripheral blood cells 8 weeks (**B**) and 16 weeks (**C**) after transplantation. (**D**) Further cell subtype analyses of the bone marrow compartment. Values are the mean ± SD, n = 9, *P < 0.05, **P < 0.01, ***P < 0.001.

Next, we sought to investigate the ability of aged OPN^−/−^ HSCs to repopulate irradiated hosts in the presence of competing WT donor cells. Peripheral blood and bone marrow samples were assayed 8 and 16 weeks following transplantation for the relative contribution of donor cells to the blood and bone marrow, based on their expression levels of CD45.1 or CD45.2 antigens (Fig. 4A). While no difference in the overall repopulation rates between the groups were detectable (Supplementary Fig. 4A), we observed a significantly lower relative contribution of peripheral blood cells (all WBC lineages) from aged OPN^−/−^ donors compared to their WT counterparts at both time points (Fig. 4B and C). Similarly, when we calculated the absolute numbers of peripheral blood cells, we detected a significant decrease in the number of mature leukocyte subsets from aged OPN-deficient bone marrow (Supplementary Fig. 4B). However, when we analyzed the primitive bone marrow subtypes, we noticed similar relative levels of immunophenotypically defined stem cells present in the recipients of both aged WT and OPN-KO donors (both groups were composed of about 75% HSCs; Fig. 4D). In contrast, the proportion of MPPs and myeloid-restricted progenitors dramatically decreased in the OPN mutant compartment, with fewer than 20% progenitor cells derived from OPN^−/−^ donors and WT controls stably contributing approximately 60% of progenitor cells (Fig. 4D). We hypothesize that the reason for the disproportionate decrease in more mature progenitor cells compared to the abundance of stem cells, as well as unaltered apoptosis and proliferation rates, is likely due to impaired differentiation from stem cells to progenitor cells in absence of OPN expression in aged animals.

## Discussion

Aging of organs with high cellular turnover is associated with a decline in the regenerative capacity of organ-specific stem cells^7,27^. The loss of stem cell function is thought to be caused by multiple factors, including genetic, epigenetic, and microenvironmental alterations^28^. We and others have previously shown that mice engineered to be deficient in molecules that regulate HSC proliferation may not only affect steady-state differentiation and the reconstitution ability of HSCs in young mice^6,25,29–31^ but may also exert effects on the reconstitution ability of aged animals^6,30^. However, little is known about age-associated changes in the composition of the stem cell microenvironment and its impact on HSC function. Here, we demonstrated that changes in the expression levels of an important component of the stem cell microenvironment, OPN, have multiple effects on HSC homeostasis in aged mice. During aging, mice lacking OPN have increased numbers of B- and T-lymphocytes and decreased numbers of erythroid cells whereas no difference in the granulocytic lineage between OPN^−/−^ and age-matched WT controls were detectable. One of the distinct hallmarks of the aging hematopoietic system is a skewing of the composition of peripheral blood cells: lymphoid cells predominate in young animals, whereas higher numbers of myeloid cells are present in aged mice^7,28^. These changes can be partially attributed to a loss of lymphoid-primed MPPs^32^. However, the preserved lymphoid cell counts of both B- and T- lymphocytes in the peripheral blood of aged OPN^−/−^ mice without affecting the cell counts of the myeloid lineage might be due to the participation of OPN in cell fate decisions at an early stage of differentiation toward lymphoid progenitors. Interestingly, in a transplant setting B-lymphocytes recover faster in the primary transplant while show a significant delay in recovery in the secondary transplant.

Osteopontin is predominantly expressed by components of bone marrow stroma and is found at virtually negligible levels within hematopoietic cells, especially the HSPC compartment. When bone marrow cells from young OPN^−/−^ mice were transplanted into WT niche the phenotype of OPN-deficiency was rescued^16^. The more surprising was here the observation that transplanting bone marrow cells from old osteopontin deficient mice resulted in a dramatic loss of reconstitution ability even in presence of OPN in the recipient mice. One could speculate that long term residence of HSC in OPN deficient microenvironment has durable effect on reconstitution ability of HSCs. Similar effect was observed when HSC have been exposed to Dkk1 expressing microenvironment opposing Wnt signaling which resulted in reduced self-renewal potential in subsequent transplants^33^.

The reconstitution ability of aged hematopoietic precursors declines during aging^7,28^. As OPN expression in the bone marrow compartment also declines in aged animals^22,23^, we investigated the reconstitution ability of HSCs from aged OPN^−/−^ mice compared to age-matched controls in two transplantation settings. In both competitive and serial transplantation assays, OPN-deficient bone marrow cells showed significantly decreased reconstitution ability compared to their aged WT counterparts. Interestingly, the effect of reduced repopulation ability was more pronounced in the presence of competitor cells, while in absence of competitor cells a sequential transplant was necessary to uncover the defect in repopulation potential of aged OPN^−/−^ bone marrow cells. Of note, under steady-state conditions, as well as during transplantation, the numbers of immunophenotypically defined stem cells did not differ between OPN^−/−^ and WT mice. However, the numbers of MPPs significantly decreased in the absence of OPN. This imbalance is consistent with our observations that OPN^−/−^ HSCs exhibit a reduced response to stimulatory cytokines in OPN-deficient HSCs (unpublished data).

The processes of cell cycle control, apoptosis, differentiation, and self-renewal are closely intertwined in regulating stem cell fate during organ homeostasis. Since OPN has been shown to alter the HSC pool by affecting apoptosis and cellular proliferation in young mice^16,17^, we investigated whether these mechanisms are also responsible for the loss of function in aged OPN^−/−^ animals. Surprisingly, we did not detect a difference in proliferation activity or rate of apoptosis between aged OPN-KO and WT HSPCs. However, there was a significant shift from G0 to G1 phase of the cell cycle in the immature bone marrow cell subsets including the highly stem cell enriched population. Stem cell dormancy and low rates of cell divisions are associated with long term repopulating ability^24,34,35^. Therefore loss of quiescence could also be accountable for the loss of repopulation ability in old OPN-deficient stem cells.

In summary, the data presented here indicate that OPN is involved in the decline of reconstitution ability of primitive HSCs during aging. OPN appears to play different roles in young and old animals. To the best of our knowledge, this is the first report on an extracellular matrix component of the bone marrow that specifically alters HSC function in an age-dependent manner.

## Methods

### Mice

OPN 129/C57BL/6^−/−^ transgenic mice were previously described^16^. These mice were backcrossed to C57BL/6 mice for at least six generations prior to use in this study. The colony was also crossed to the congenic strain Bl6-SJL CD45.1 to obtain CD45.1 or CD45.1&45.2 heterozygous offspring for transplantation experiments. Mice heterozygous for the OPN transgene (OPN^+/−^) were mated to obtain littermates with the following genotypes: WT, OPN^−/−^, or OPN^+/−^.

All animal experiments were approved by the federal office for Nature, Environment and Consumer Protection, North Rhine Westphalia (Landesamt für Natur, Umwelt und Verbraucherschutz NRW) and local authorities at the University of Bonn (Protocol # 84–02.04.2011.A330). All experiments were performed in accordance with relevant guidelines and regulations. The mice were housed at the University of Bonn animal facility (Haus für Experimentelle Therapie) according to institutional guidelines. They were sacrificed by increasing CO2 concentrations, as recommended by the corresponding authorities.

### Automated peripheral blood analysis

Peripheral blood was obtained by tail vein nicking, collected in EDTA-coated containers (Microtainers; BD Biosciences, Franklin Lakes, NJ, USA), and measured within a few hours of collection. Total numbers of WBCs, erythrocytes, and platelets were determined using a Hemavet 950 Analyzer (Drew Scientific, Dallas, TX, USA).

### Sample collection and flow cytometric analysis

#### Blood

Anticoagulated whole blood was subjected to erythrocyte lysis; the remaining cells were fixed in FACS lysis buffer (BD Biosciences) according to the manufacturer’s instructions. After washing with staining buffer [SB; PBS containing 1% fetal calf serum Gold (PAA Laboratories GmbH, Cölbe, Germany)], the cells were stained with antibodies specific for mature blood cell surface markers: B220-APC-eF780 for B lymphocytes, CD3e-PE-Cy7 for T lymphocytes, Gr1-eF660 for granulocytes, and Mac1α-eF450 for monocytes. Anti-CD45.1-PE and anti-CD45.2-FITC antibodies were used to discriminate congenic strains. All antibodies were purchased from eBioscience (San Diego, CA, USA); reactivity was against mouse antigens unless stated otherwise.

### Bone marrow

Bone marrow was freshly prepared from the hind legs of a sacrificed mouse and washed with phosphate buffered saline (PBS) supplemented with 0.5% fetal calf serum. The cells were stained with the antibodies previously described, and flow cytometry was performed on a BD FACS Canto^TM^ II flow cytometer (BD Biosciences). Briefly, the samples were re-suspended in SB and centrifuged; the pellet was re-suspended in the corresponding antibody mixture and incubated at either room temperature or 4 °C for 20 min. Excess antibody was removed by the addition of 1 mL SB to each sample, followed by centrifugation. Finally, cells were re-suspended in an adequate volume of SB and analyzed by FACS.

To identify HSC-containing populations, mature bone marrow cells were excluded by staining with a cocktail of biotinylated anti-mouse antibodies to Mac-1a (CD11b), Gr-1 (Ly-6G and Ly-6C), Ter119 (Ly-76), CD3, and B220 (CD45R), followed by subsequent staining with streptavidin-conjugated eFluor 450 or PerCP. Additionally, c-Kit-APC, Sca1-PE-Cy7 (Ly 6A/E), CD150-Alexa Fluor 488 (BioLegend, San Diego, CA, USA), and CD48-PE were used to discriminate the different bone marrow cell subtypes.

To assess the phase of the cell cycle in primitive cell populations, a single intraperitoneal injection of BrdU (2 mg/animal) was administered to each animal. All animals additionally received 1 mg/mL BrdU (Sigma) in drinking water for approximately 14 h for the BrdU incorporation assay. Bone marrow cells were stained with lineage antibodies, antibodies specific for c-Kit, Sca-1, CD150, and CD48 (as described above), and BrdU. For the latter, cells were fixed and permeabilized using the BrdU Flow kit (BD Biosciences) according to the manufacturer’s instructions. Nuclei were counterstained with DAPI (1:2,000).

For apoptosis analysis, we stained cells for the surface markers described above and annexin V-FITC (BD Biosciences) according to the manufacturer’s protocol. Nuclei were counterstained with DAPI (1:2,000) to assess cell viability.

### Transplantation assays

For transplantation assays, a congenic mouse system was utilized: donor cells expressed the pan-hematopoietic surface marker CD45.1 or CD45.1&2 and were transplanted into CD45.2-bearing hosts. WT female C57BL/6 recipients were obtained from Charles River Laboratories. WT CD45.1&2 and OPN^−/−^ mice were bred in-house. Engraftment efficiency in recipients was monitored by donor contribution of CD45.1-positive cells using FACS analysis. Peripheral blood cell analyses were performed at different time points after transplantation to assess short- and long-term reconstitution ability.

For serial transplantation, eight weeks after the first round of transplantation, recipients were used as donors for the next transplantation cycle to assess the self-renewal ability of repetitive transplants. Transplants were discontinued after the third round of transplantation due to a lack of peripheral blood reconstitution in the third-round recipients of aged bone marrow.

For the competitive reconstitution assay, 1 × 10^6^ cells from whole bone marrow of 8–12-week-old WT CD45.1 (competitor) mice were mixed with the same amount of cells from whole bone marrow of age-matched (22 months of age) CD45.1&CD45.2 WT or OPN^−/−^ mice and injected into lethally irradiated eight-week-old CD45.2 recipient mice. Reconstitution was assessed by flow cytometry at different time points after transplantation for multi-lineage reconstitution as indicated.

### Molecular analyses

For qPCR analyses RNA was isolated from total tibia lysates using the Trizol Reagent (Invitrogen), reverse transcribed using the high capacity cDNA transcription kit (life technologies). The qPCR was carried out using the TaKaRa Syber Premix Ex Taq-kit on an Eppendorf Mastercycler epRealplex machine. qPCR analyses have been established following the MIQE guidelines^36^ applying 3 reference genes for callibrating the relative expression level. Primer-sequences are listed in supplementary Table S1.Data was analysed using the qBase Plus software (Biogazelle). Results are expressed as a ratio of the mean expression level in OPN deficient cells compared to the WT controls. Statistical analysis was done using the unpaired two-tailed student t-test.

### Statistical analysis

Statistical analysis was performed using the unpaired two-tailed Student’s *t* test; significance was defined as a *P* value of less than 0.05.

## Electronic supplementary material

Supplementary Figures and Table
