# Supplementary material for: Murine hematopoietic stem cell reconstitution potential is maintained by osteopontin during aging
Source: Sci Rep. 2018 Feb 12;8:2833. doi: 10.1038/s41598-018-21324-x (PMC5809550; doi:10.1038/s41598-018-21324-x)
Supplement: Supplementary file 1 — Supplementary Figures and Table [file 41598_2018_21324_MOESM1_ESM.pdf]

Supplemental Figures

Murine hematopoietic stem cell reconstitution potential is maintained by osteopontin during aging

Jin Li, Carmen Carrillo García, Tamara Riedt, Maria Brandes, Sabrina Szczepanski, Peter Brossart, Wolfgang Wagner, and Viktor Janzen

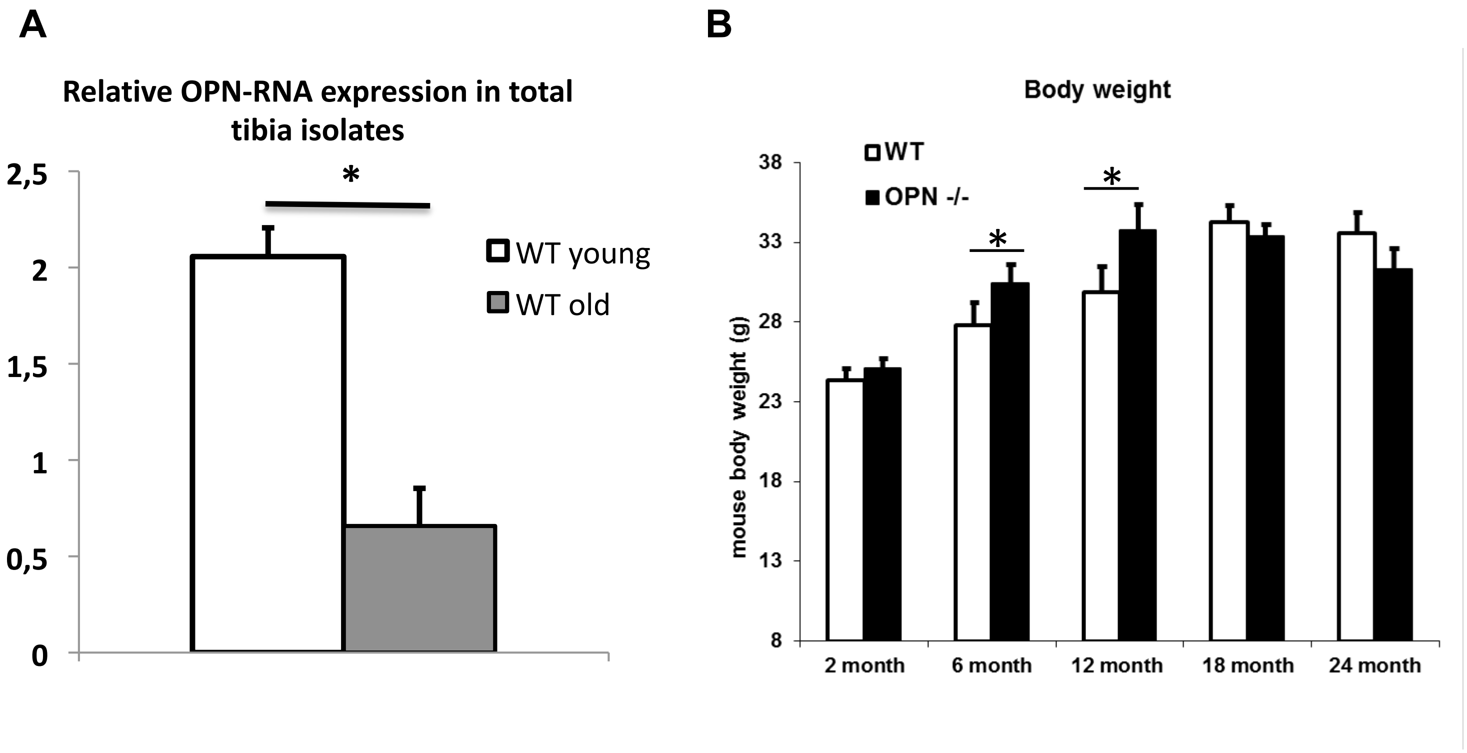

Supplementary Figure 1: OPN expression declines with age but OPN deficiency does not alter body weights or bone marrow cellularity of OPN-/- animals. (A) OPN-mRNA expression was analyzed by qPCR in total tibia isolates and is reduced in 22 month old mice compared to 2 month old mice. (B) Mice were monitored for changes in body weight over a course of two years. No differences in body weight were detectable between old WT and $OPN^{-/-}$ animals. Values are the mean ± SEM; n ≥ 12; *$P \leq 0.05$.

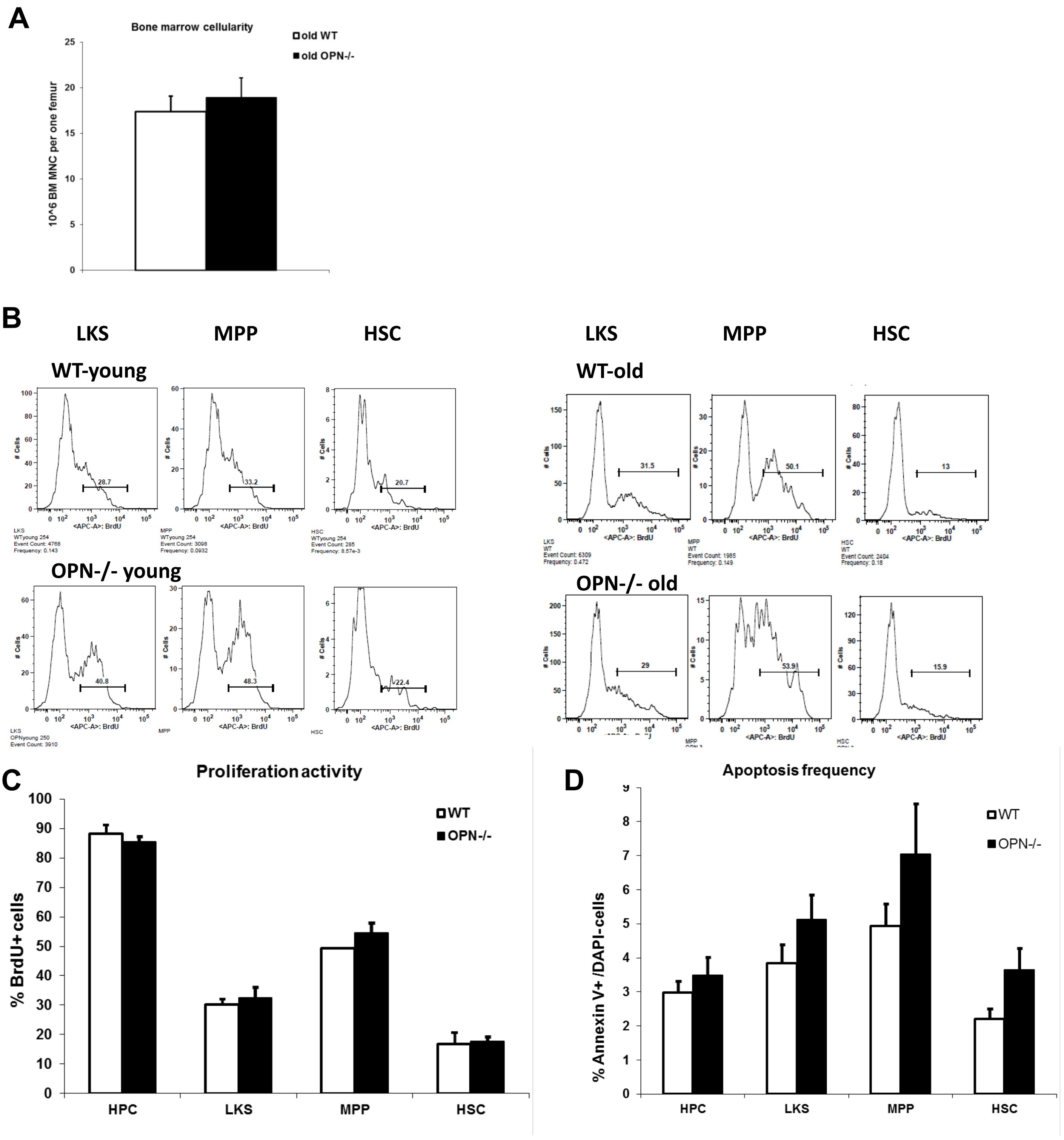

Supplementary Figure 2: OPN deficiency does not alter the cell cycle or apoptosis in aged HSPC subtypes. (A) no difference in bone morrow cellularity in 20-24 month old mice. (B) Representative FACS plots of the BrdU incorporation assay of young and aged cell subsets. (C) Cumulative data of proliferation activity in HSPC subpopulations. (D) No significant differences in the rate of apoptotic cells between old WT and OPN-/- HSPC subpopulations were detectable. Values are the mean ± SEM; n ≥ 5; **P* = ns.

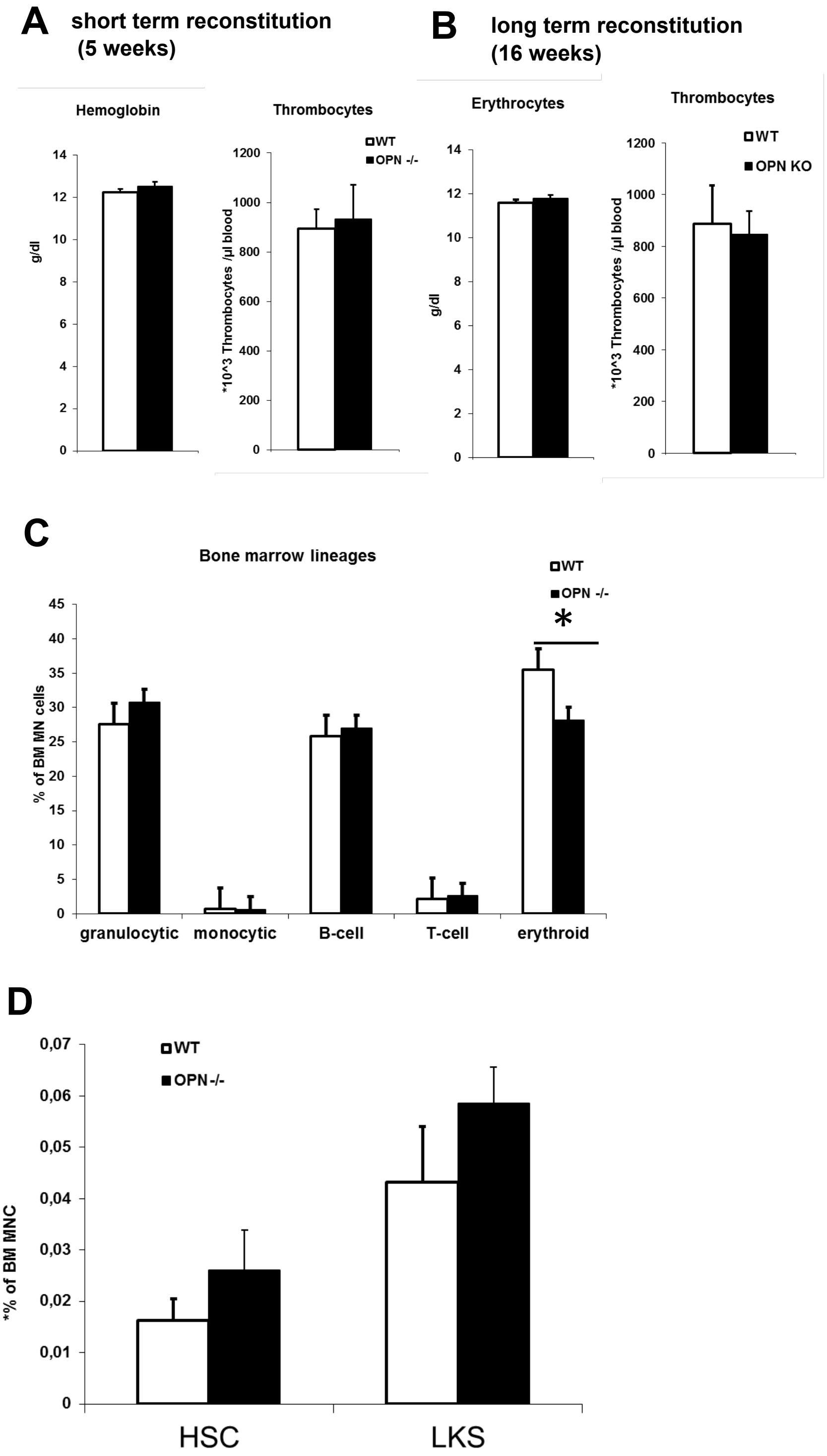

Supplementary Figure 3: Impact of OPN deficiency on repopulation ability. (A-B) Comparable short term and long term repopulation of peripheral blood erythrocytes and thrombocytes in the first transplant round. (C) Bone marrow lineage analyses of hematopoietic reconstitution 16 weeks after transplantation revealed reduced erythroid progenies in recipients of old OPN-/- bone marrow. Values are the mean ± SD; n = 5; $^*P \leq 0.05$. (D) Recipients of the second round of serial bone marrow transplant with aged WT and OPN-deficient bone marrow cells display no significant difference in stem and progenitor frequencies 16 weeks after transplant. Values are the mean ± SD; n = 8; *P* =n.s.

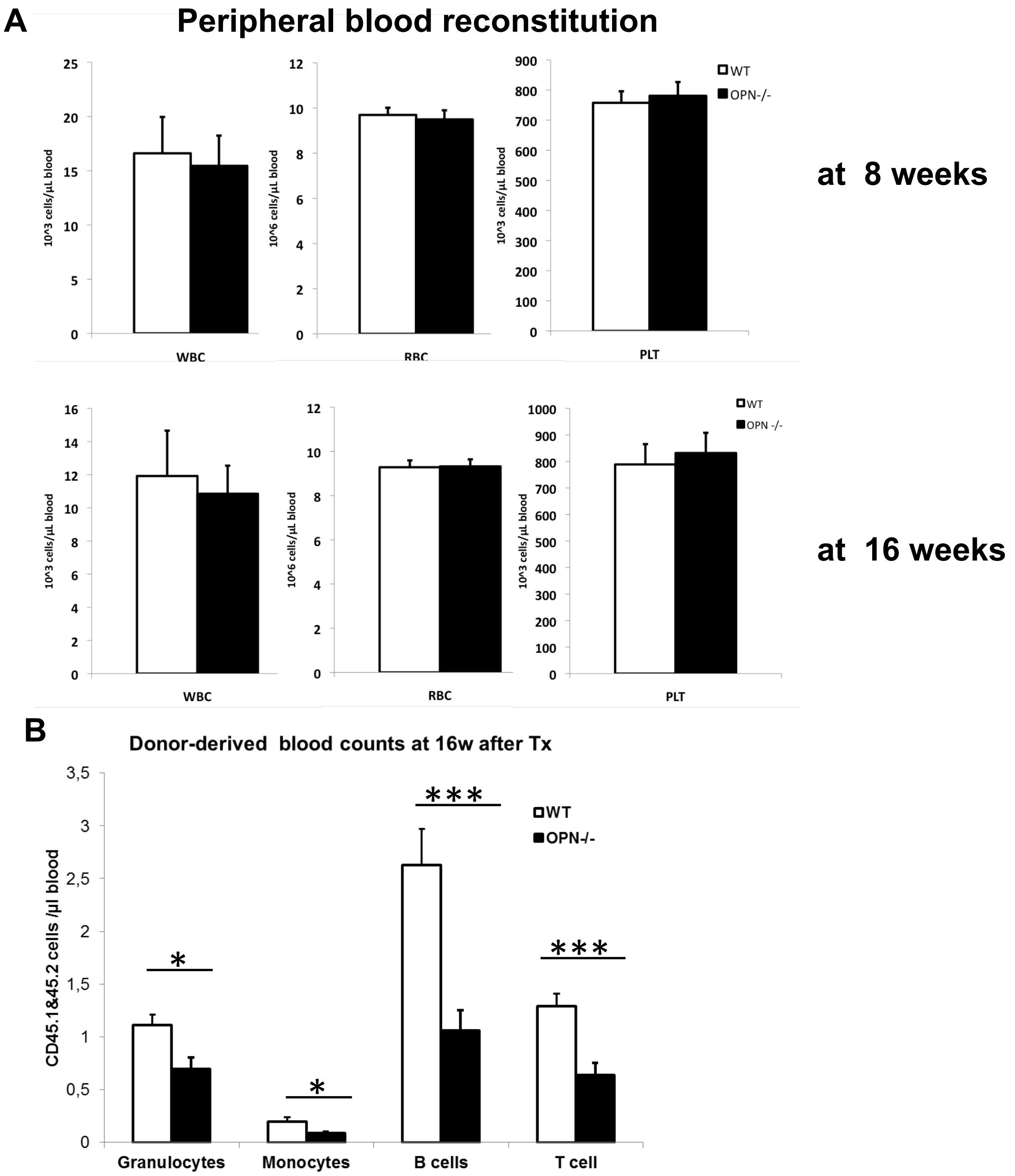

Supplementary Figure 4: Aged OPN-deficient bone marrow cells display a reduced reconstitution ability in a competitive transplantation setting. (A) Peripheral blood cell counts (composed of test and competitor derived cells) on white blood cells (WBC), red blood cells (RBC), and platelets (PLT) show no difference in repopulation rates between the recipients of old WT or OPN-/- bone marrow in a competitive transplant. (B) Calculation of test donor-contributed cells from aged WT and OPN-/- animals per µL peripheral blood. Values are the mean ± SD; n = 8; $^{*}P \leq 0.05$, $^{***}P \leq 0.001$.

Supplementary Table S1.
Primer sequencies for qPCR

Oligoname	Sequence 5' -> 3'
β-Actin-F (reference gene)	AGTGTGACGTTGACATCCGTA
β-Actin-R (reference gene)	GCCAGAGCAGTAATCTCCTTCT
GAPDH-F (reference gene)	AGGTTGTCTCCTGCGACTTCA
GAPDH-R (reference gene)	GGTGGTCCAGGGTTTCTTACTC
TBP-F (reference gene)	TCTATTTTGGAAGAGCAACAAAGAC
TBP-R (reference gene)	GAGGCTGCTGCAGTTGCTA
YWHAZ-F (reference gene)	AGACGGAAGGTGCTGAGAAA
YWHAZ-R (reference gene)	TCAAGAACTTTTCCAAAAGAGACA
OPN-F	CGTCCCTACAGTCGATGTCC
OPN-R	TGACTCATGGCTGCCCTTTC
